# Unraveling the microRNA of *Caragana korshinskii* along a precipitation gradient on the Loess Plateau, China, using high-throughput sequencing

**DOI:** 10.1371/journal.pone.0172017

**Published:** 2017-02-16

**Authors:** Pengbo Ning, Yulu Zhou, Lifang Gao, Yingying Sun, Wenfei Zhou, Furong Liu, Zhenye Yao, Lifang Xie, Junhui Wang, Chunmei Gong

**Affiliations:** 1 College of Life Science, Northwest A&F University, Yangling, Shaanxi, China; 2 School of Life Science and Technology, Xidian University, Xi’an, Shaanxi, China; Henan Agricultural University, CHINA

## Abstract

Drought remains one of the main factors that negatively affect plant growth and development. *Caragana korshinskii* is widely distributed on the Loess Plateau, China, where it mediates soil and water loss and helps prevent desertification. However, little is known about the stress response mechanisms of *C*. *korshinskii* in water-starved environments. MicroRNAs (miRNAs) have been implicated in the regulation of plant responses to several types of biotic and abiotic stress. Here, we describe the miRNAs of wild *C*. *korshinskii* from Huangling, Yulin, and Dalad Banner, which occur along a precipitation gradient. Using next-generation sequencing technology, we obtained a total of 13 710 681, 15 048 945, and 15 198 442 reads for each location, respectively; after filtering and BLAST analysis, 490 conserved miRNAs and 96 novel miRNAs were characterized from the sRNAome data, with key functions determined using Gene Ontology and Kyoto Encyclopedia of Genes and Genomes pathway analyses. We also designed stem-loop qRT-PCR to validate the expression patterns of 5 conserved miRNAs (miR390, miR398, miR530, miR2119, and miR5559) that obviously responded to water stress in plants grown both under natural and experimental water stress conditions and found that the expression levels of miR2119 and miR5559 were negatively correlated with their predicted target genes. This study is the first to identify miRNAs from wild *C*. *korshinskii* and provides a basis for future studies of miRNA-mediated gene regulation of stress responses in *C*. *korshinskii*.

## Introduction

MicroRNAs (miRNAs) are small, non-coding RNAs of ~22 nucleotides (nt) in length that play pivotal regulatory roles in both animals and plants [1]. In fact, miRNAs are indispensable players in many types of gene regulation and function; they act by binding to complementary messenger RNA (mRNA) from specific target genes, thus causing mRNA degradation and gene silencing [2]. In addition, miRNAs constitute the majority of gene regulatory molecules involved in regulatory pathways and influence the output of a variety of protein-coding genes. A large amount of evidence also indicates that miRNAs are involved in various aspects of plant growth and development, such as leaf morphogenesis [3], phase transition [4], and adaptive responses to abiotic and biotic stress [5,6].

Among the many types of environmental stress, drought remains one of the most important factors limiting plant growth and development. For example, the Loess Plateau, which is located in the semi-arid regions of northwest China, is a fragile ecosystem that is affected by drought, as well as by other natural factors. Global climate change has recently heightened drought in this region, and both grassland degradation and desertification now threaten to destroy the ecosystem [7,8]). Therefore, investigating drought-resistant plants of the Loess Plateau may reveal effective ways to prevent the spread of desertification, which can then be used to facilitate the long-term protection of the ecosystem’s balance and stability.

*Caragana korshinskii* (Fabaceae), widely known as a “life-saving plant,” is a perennial leguminous shrub that is distributed in the arid and semi-arid regions of northwest China [9,10]. It is a high-quality forage plant, perfectly adapted to drought stress. It is also a pioneer plant and functions to control soil erosion and desertification of the grasslands of the Loess Plateau. Investigating the molecular mechanisms of the plant’s drought resistance is still an attractive and popular target for research, owing to its potential uses in agricultural production, vegetation rehabilitation, and ecological restoration [11]. However, few studies have investigated the contribution of miRNA to the adaptive mechanisms of *C*. *korshinskii*.

In recent years, the HiSeq high-throughput sequencing technology has been widely applied to investigating novel species-specific or low-abundance miRNAs [12,13]. The technology has been applied to multiple model plants such as rice [12] and tobacco [14], as well as non-model plants, such as Chinese yew [15], peanut [16], and cowpea [17].

We used high-throughput sequencing to analyze the miRNA expression profile of wild *C*. *korshinskii* along a precipitation gradient on the Loess Plateau, China. In addition, we aimed to determine the significance of known and novel miRNAs from their expression profiles and to predict their target genes. The present study presents significant evidence for the role of miRNAs in the drought-tolerance of *C*. *korshinskii*. Further characterization of the targets of drought-associated miRNAs will help elucidate the response and tolerance of *C*. *korshinskii* to drought.

## Materials and methods

### Sampling sites, conditions, and plant material

**Fig 1:**
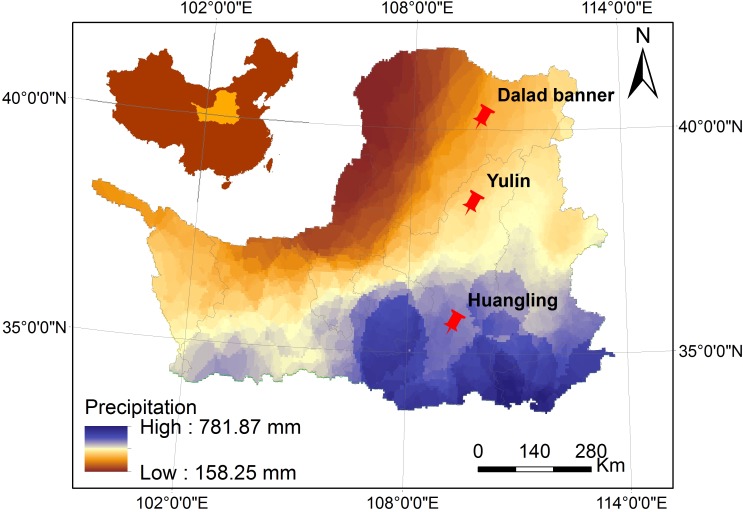
Three different sampling sites of *Caragana korshinskii* on the Loess Plateau, China.

The Loess Plateau is located in the continental monsoon region of China, where the annual precipitation forms a natural gradient from 150 mm in the northwest to 800 mm in the southeast; the majority of the rainfall is concentrated between June and September [18], and the annual evaporation ranges from high in the northwest to low in the southeast [19,20]. Three *C*. *korshinskii* experimental sites, Huangling (HuL; 35°39'N,109°14'E), Yulin (YuL; 38°19'N, 109°43'E) and Dalad Banner (DaB; 40°14'N, 109°59'E), were selected along the precipitation gradient across Shaanxi Province and Inner Mongolia, Northwest China (Fig 1). As shown in our previous study of *C*. *korshinskii* [21], the annual precipitation, annual evaporation, soil moisture content, and plant leaf water potential of the sites differ significantly, and the positive correlations that leaf water potential shows with annual precipitation (R = 0.968, P < 0.01) and soil moisture content (R = 0.963, P < 0.01) indicate that water stress increases gradually from the south to the north. Moreover, the annual average temperature from the northwest to the southeast, and the soil pH and altitude along the precipitation gradient are known to be similar [22].

Three circular plots (20 m radius) were established at each site, and both plant and soil samples were collected from each site in July 2013. All of the sampled *C*. *korshinskii* shrubs were over 20 years old. Leaf samples (~5 g) were collected, and their water potential was determined using a WP4-T dew-point meter (Decagon Devices, Pullman, WA, USA). Upper mature leaflets with healthy extensions were immediately frozen in liquid nitrogen, transported to the laboratory, and stored at -80°C prior to enzyme activity analysis and total RNA isolation.

We state that no specific permissions were required for these locations/activities. We confirm that the field studies did not involve endangered or protected species.

### Small RNA library construction and sequencing

To construct the small RNA (sRNA) library, all three samples were pooled, and total RNA was extracted from the mixture according to the manufacturer’s instructions. About 10 mg of sRNA was used for sequencing. The sRNA fractions with lengths of 10–40 nt were isolated using 15% denaturing polyacrylamide gel electrophoresis, ligated to 5′ and 3′ adaptors, and reverse-transcribed to cDNA, according to the Illumina protocol. The resulting cDNA libraries were then sequenced by the GENE DENOVO Company (China).

### miRNA identification

The raw sequences were first processed using Illumina’s Genome Analyzer Pipeline software in order to filter out the adapter sequences, as well as low quality and low-copy sequences. Then, RNA sequences of 18–30 nt in length were mapped to transcriptome data (https://www.ncbi.nlm.nih.gov/sra/?term=SRX1117100), RFam (http://rfam.xfam.org/), and Repbase (http://www.girinst.org/repbase/), in order to identify and filter out mRNA, rRNA, tRNA, snRNA, snoRNA, and repeat sequences. Finally, the remaining unique sequences were compared to the database of published miRNA sequences, miRBase 21 (http://www.mirbase.org/) using BLASTn search to identify conserved miRNAs. A maximum of three mismatches was allowed between the miRNAs of *C*. *korshinskii* and those from other plant species, and only perfectly matched sequences were considered conserved [23]. small RNA tags that were not identified from the public databases were aligned with the reference genome. According to their genome positions and hairpin structures predicted by the software MIREAP (version 0.2, http://sourceforge.net/projects/mireap/;) [24], the novel miRNA candidates were identified [25,26].

### Target prediction and functional annotation

The target genes of the known miRNAs were predicted as those occurring at the intersection of the MIREAP, miRanda, and TargetScan results. The criteria used for target prediction were based on those suggested by Allen et al. [25] and Schwab et al. [26]: (1) no more than 4 mismatches between the sRNA and the target (G-U bases count as 0.5 mismatches); (2) no more than two adjacent mismatches in the miRNA/target duplex; (3) no adjacent mismatches in positions 2–12 of the miRNA/target duplex (5' of miRNA); (4) no mismatches in positions 10–11 of the miRNA/target duplex; (5) no more than 2.5 mismatches in positions 1–12 of the miRNA/target duplex (5' of miRNA); and (6) minimum free energy of the miRNA/target duplex should be ≥75% of the minimum free energy of the miRNA bound to its perfect complement.

In order to better understand miRNA target function and classification, as well as the metabolic regulatory networks associated with *C*. *korshinskii* miRNAs and their targets, Gene Ontology (GO; http://www.geneontology.org/) enrichment analysis and Kyoto Encyclopedia of Genes and Genomes (KEGG) pathway analysis (http://www.genome.jp/keg/) were performed for the target genes, as described previously [27].

### Verification of miRNAs by quantitative real-time PCR (qRT-PCR)

Stem-loop qRT-PCR was carried out to validate the presence and expression of the identified miRNAs. Total RNA was extracted from leaves using Trizol (TaKaRa, Dalian, China), according to the manufacturer’s instructions, and the RNA concentrations and integrity were analyzed using a NanoDrop 2000 (Thermo Fisher Scientific, Waltham, MA, USA) and agarose gel electrophoresis. Finally, the RNA was treated with RNase-free DNase I (TaKaRa) to remove genomic DNA. Sequence-specific forward primers were designed for 9 selected miRNAs (S1 Table), and qRT-PCR was performed using SYBR RealMasterMix (Tiangen Biotech, Beijing, China) and the ABI 7300 real-time PCR detection system (Applied Biosystems, USA), with the following amplification conditions: denaturation at 95°C for 3 min followed by 40 cycles of denaturation at 95°C for 15 s and annealing and extension at 61°C for 30 s. Finally, melting analyses were performed to confirm the absence of false-positive peaks. All reactions were performed in triplicate for each sample, and U6 snRNA was used as an internal reference [28]. The relative expression levels of the miRNAs were calculated using the 2^-ΔΔCt^ method [29].

### Drought treatment of *C*. *korshinskii* in laboratory

*C*. *korshinskii* seeds were obtained from the Loess Plateau and allowed to germinate at 28°C in the dark. After 2 d, the seeds were individually sown in pots (11 × 35 cm) filled with a 1:1 (v:v) mixture of rich soil and vermiculite and then kept in a growth chamber with a 16-h light/8-h dark photoperiod and 25/18 (±2) °C day/night temperature cycle [30]. After 3 months, when the seedlings had grown to ~25 cm, the plants were assigned to one of three soil moisture levels (75%, 55%, 35%), which corresponded to suitable water availability (CK), mild drought stress (MD), and severe drought stress (SD), respectively [31]. The soil moisture levels were then maintained constant for 20 d, as described previously [32]. After the drought treatment, leaf samples were collected, frozen in liquid nitrogen, and stored at -80°C. Three replicates were performed for each treatment.

### Statistical tests

All data were analyzed using one-way ANOVA in SPSS 17.0 (SPSS Inc., Chicago, IL, USA), with differences of P < 0.05 considered statistically significant. Experimental data are reported as mean values ± SE (n = 3).

## Results

### Small RNA library sequence analysis

The miRNA of *C*. *korshinskii* leaves collected along a precipitation gradient on the Loess Plateau, China, were characterized using HiSeq Solexa SBS technology. We obtained 13 710 681, 15 048 945, and 15 198 442 total reads; and 13 705 945, 15 043 247, and 15 192 717 high-quality reads after filtering for the YuL, HuL, and DaB libraries, respectively. After removing contaminant reads, we identified 13 620 247, 14 954 392, and 15 102 242 clean tags from the three locations, respectively (Table 1). These clean reads were annotated to 490 known miRNA families (S2 Table), and 96 novel miRNAs were predicted from the remaining unannotated tags (11 655 769 clean reads).

**Table 1:** Statistics of small RNA sequences from the *Caragana korshinskii* libraries.

Category	HuL		YuL		DaB	
	Count	Percent (%)	Count	Percent (%)	Count	Percent (%)
**total_reads**	13710681		15048945		15198442	
**high_quality**	13705945	100%	15043247	100%	15192717	100%
**3'adapter_null**	23996	0.18%	27573	0.18%	22424	0.15%
**insert_null**	16067	0.12%	14945	0.10%	20034	0.13%
**5'adapter_contaminants**	7754	0.06%	7642	0.05%	8250	0.05%
**smaller_than_18nt**	34563	0.25%	34190	0.23%	36983	0.24%
**polyA**	3318	0.02%	4505	0.03%	2784	0.02%
**clean_reads**	13620247	99.37%	14954392	99.41%	15102242	99.40%

**Fig 2:**
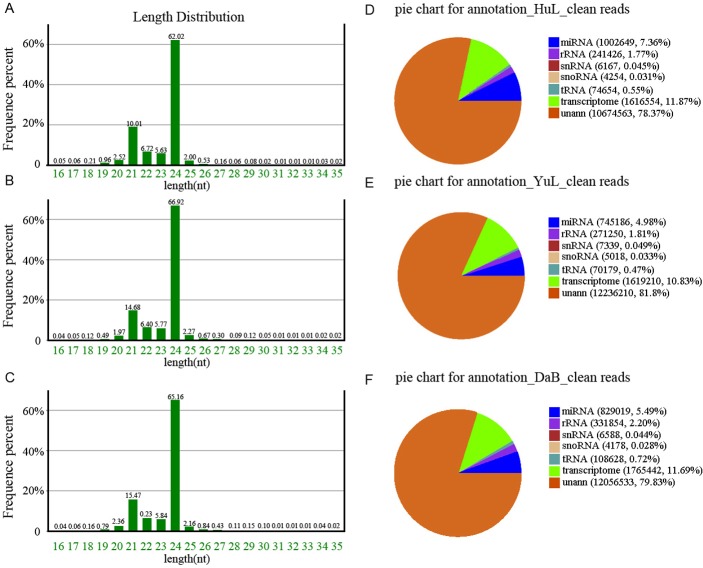
Summary of cleaning reads and length distribution of tags produced by small RNA sequencing in three locations. (A-C) Length distribution of miRNA in the three libraries. The x-axis represents the length of the small RNA. The y-axis represents the percent of distribution. (D-F) Summaries of annotated as noncoding RNAs and unannotated miRNA. A and D are the samples from Huangling (HuL); B and E are the samples from Yulin (YuL), and C and F the samples are from Dalad Banner (DaB).

The majority of the small RNAs were 20–24 nt in length, with the 24-nt class being the most abundant (64.7%), followed by the 21-nt class (16.35%); many of the small RNAs were annotated as noncoding RNAs (Fig 2). We easily notice all kinds of small RNA, in which the unannotated miRNA accounts for a proportion of the unique RNA, approximately 80%, miRNA accounts for proportion just 5–7% in total sRNAs. These distribution patterns are typical of small RNA distributions in plants such as *Medicago truncatula* [33] and peanut [34].

### Identification of conserved and novel miRNAs

**Fig 3:**
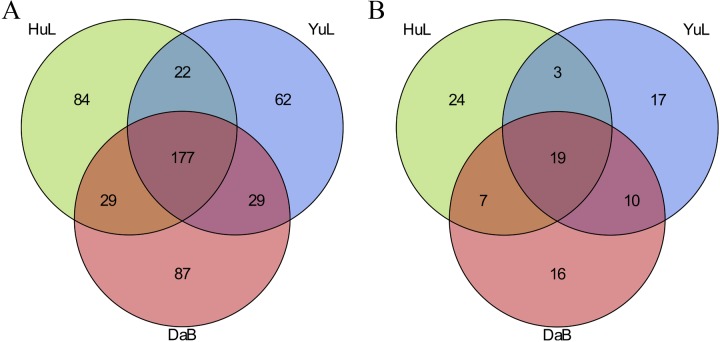
Venn diagrams represent a comparison of known and novel miRNAs among three data sets. The number marked in the overlapping areas indicates the common miRNAs. (A) Number of known miRNAs. (B) Number of novel miRNAs.

A total of 490 known miRNAs were identified from the three libraries; the HuL, YuL, and DaB sites had 312, 290, and 321 miRNAs, respectively (S2 Table). Of these, 84 (17.14%), 87 (17.76%), and 62 (12.65%) of the miRNAs were unique to the HuL, YuL, and DaB libraries, respectively (Fig 3A). Moreover, HuL-YuL shared 199, YuL-DaB shared 206, and HuL-DaB shared 206 miRNAs, while 177 miRNAs (36.12% of 490 miRNAs) were common to all three. sites. Regarding the unannotated sequences, 96 candidate novel miRNAs were predicted (S3 Table), among which 19 novel miRNAs (19.79% of 96 novel miRNAs) were occurred in all three sites (Fig 3B).

**Fig 4:**
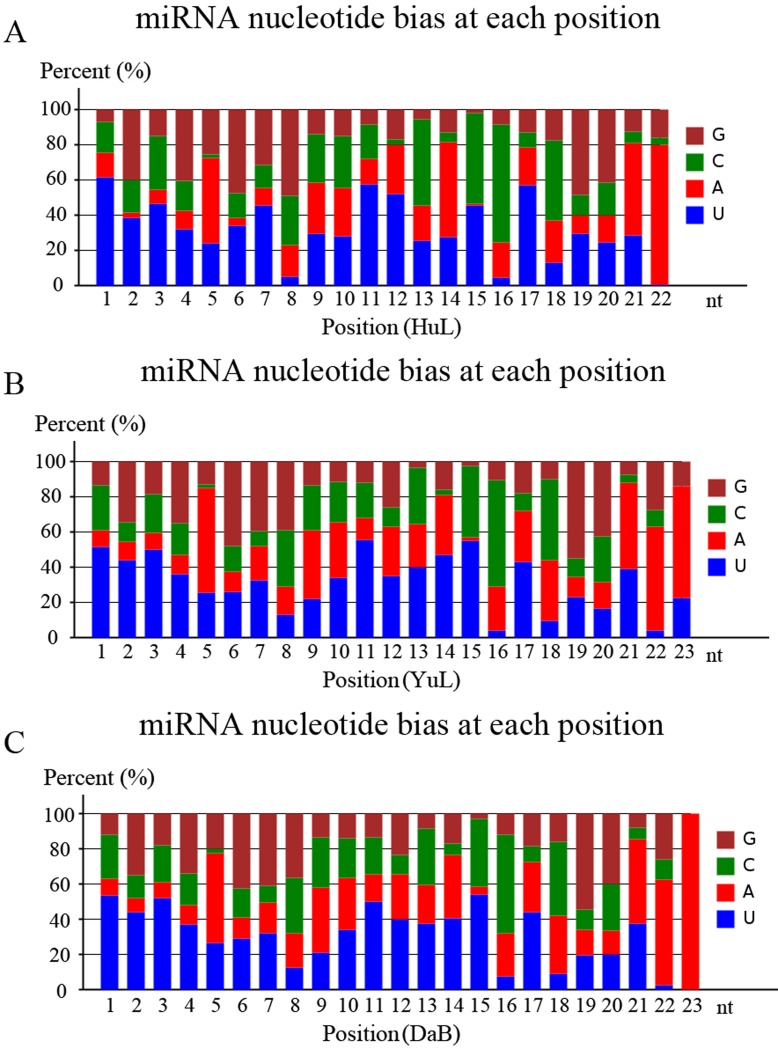
Nucleotide bias of novel miRNAs from three *Caragana korshinskii* cDNA libraries. Nucleotide bias at each position of novel miRNAS from the Huangling (HuL) library (A), Yulin (YuL) library (B), and Dalad Banner (DaB) library (C).

The 96 miRNAs ranged from 20 to 23 nt in length, among which 21-nt sequences were the most common (73.96%), followed by 22-nt (19.79%), 20-nt (3.13%), and 23-nt (3.13%) sequences. According to the statistical novel miRNA nucleotide bias at each position, we were able to evaluate the accuracy of the novel miRNA predictions. We found that up to 50% of the novel predicted miRNAs possessed 5′ U bases, and A bases were observed at a high frequency in the 10^th^ and 11^th^ base positions (Fig 4).

### Identification of drought stress-related miRNAs

In the present study, 2298 unigene sequences were predicted as the targets of 75 conserved miRNAs, and 1274 unigene sequences were predicted as the targets of 18 novel miRNAs (S4 Table). The predicted target transcripts were known to be involved in a variety of stress-related metabolic processes, including apoptotic processes, the ER-nucleus signaling pathway, oxidoreductase activity, fatty acid metabolic processes, leaf development and the biosynthesis of jasmonic acid, oxidative stress tolerance, leaf morphogenesis, xylem and phloem pattern formation, and the auxin-activated signaling pathway. However, considering our focus on drought stress, we carefully selected 39 miRNAs for the GO and KEGG analyses, in order to elucidate the key drought-resistance mechanisms of *C*. *korshinskii* from the Loess Plateau (Table 2).

**Table 2:** Key miRNAs differentially expressed depending on water availability and their putative targets.

miRNA family	Putative Target	Function class
**MIR1533**	zinc finger CCCH domain containing protein	cytoplasmic part cation binding; mRNA binding; protein binding translation.
	SOS2 protein kinase	protein serine/threonine kinase activity; auxin polar transport; hyperosmotic response.
	Ethylene responsive transcription factor 3	DNA binding response to acid; response to stress.
**MIR2119**	bax inhibitor 1	fatty acid metabolic process; apoptotic process; response to stress; ER nucleus signaling pathway.
**MIR2645**	heat shock 70 kDa protein 16	oxidoreductase activity, acting on the CHCH group of donors; NAD or NADP as acceptor; response to stimulus.
**MIR2666**	TIRNBSLRR RCT1 resistance protein	response to stimulus.
**MIR319**	transcription factor GAMYB isoform X1	response to hormone; programmed cell death; regulation of cellular process.
**MIR3629**	heat shock 70 kDa protein 17	Protein processing in endoplasmic reticulum plastid; oxidoreductase activity,acting on the CHCH group of donors, NAD or NADP as acceptor; response to stimulus.
	abscisic acid receptor PYL8	phosphatase inhibitor activity; regulation of phosphoprotein phosphatase activity; abscisic acid activated signaling pathway; Plant hormone signal transduction.
**MIR390**	TAS3	Auxin mediated transcriptional activation.
**MIR3946**	auxin response factor 18	protein binding; phyllome development; floral organ development; root development; hormone mediated signaling pathway.
	WRKY transcription factor	DNA binding; nucleic acid binding; transcription factor activity.
	zinc finger CCCH domain containing protein 32	cation binding.
**MIR3948**	auxin regulated protein	Membrane.
**MIR394**	F-box only protein 6	leaf morphogenesis; xylem and phloem pattern formation; auxin activated signaling pathway.
	DNA directed III subunit RPC2	Purine metabolism; Pyrimidine metabolism.
**MIR395**	uncharacterized protein LOC100809991	protein kinase activity.
	mitochondrial inner membrane protease ATP23	endopeptidase activity protein processing.
**MIR397**	laccases	oxidoreductase activity, acting on diphenols and related substances as donors, oxygen as acceptor lignin metabolic process.
**MIR398**	Oxidative stress	Response to oxidative stress.
**MIR408**	replication protein A	Nucleotide excision repair; DNA replication; Mismatch repairorganic cyclic compound binding.
**MIR4415**	zinc finger protein 36, C3H1 type	DNA-binding.
	E3 ubiquitin protein ligase	Ubiquitin mediated proteolysis; membrane transition; metal ion binding; proteasome mediated ubiquitin dependent protein catabolic process.
**MIR472**	auxin response factor	binding to auxin response elements.
**MIR5175**	BI1 protein	fatty acid metabolic process; apoptotic process; response to stress; ER nucleus signaling pathway.
**MIR5232**	chlorophyll a/b binding protein	Photosynthesis antenna protein splastid envelope; chloroplast thylacation binding; generation of precursor metabolites and energy.
**MIR5244**	WRKY transcription factor	DNA binding; transcription factor activity.
**MIR5245**	Ethylene responsive transcription factor WIN1	DNA-binding; response to stress.
	auxin induced protein 5NG4	Plant hormone signal transduction.
**MIR5290**	zinc finger CCCH domain containing protein ZFN	cytoplasmic partcation binding; mRNA binding; protein bindingtranslation.
**MIR5293**	DELLA protein GA-I	Plant hormone signal transduction; response to lipid; cellular response to organic substance.
**MIR5296**	jasmonate Omethyl transferase	Sadenosy lmethionine dependent methyltransferase activity; response to stress; alphaLinolenic acid metabolism.
	zinc finger CCCH domain containing protein 48	DNA-binding.
**MIR5298**	AP2 ethylene responsive transcription factor	response to acid; response to abiotic stimulus; response to stress.
	cell division control protein	Ribosome biogenesis in eukaryotes.
	Myb DNA binding protein BAS1	nucleic acid binding.
**MIR529**	squamosa promoter binding protein	flower development; vegetative phase change.
	peroxygenase 4	response to water deprivation.
	auxin responsive protein IAA26	hormone mediated signaling pathway.
**MIR5302**	peroxisome biogenesis protein 1	Peroxisome nucleic acid binding; coupled fatty acid catabolic process; protein targeting to peroxisome.
**MIR530**	zinc knuckle (CCHC type) family protein	DNA-Binding.
**MIR535**	Zinc finger CCCH domain containing protein	DNA-Binding.
**MIR5559**	protein HIRA	intracellular membrane bounded organelle.
**MIR5658**	F box/kelch repeat protein SKIP11 isoform X1	Microbody ligase activity; metabolic process.
	protein auxin SIGNALING F box 3	floral organ development; hormone mediated signaling pathway; pollen development; postembryonic root development.
	transcription factor MYB39 isoform X2	response to hormone; cell morphogenesis involved in differentiation; response to acid; response to osmotic stress.
	NAC domain containing protein 43	DNA binding; phenylpropanoid biosynthetic process; planttype cell wall biogenesis.
	auxin response factor 9	protein binding hormone mediated signaling pathway.
	WRKY transcription factor	nucleic acid binding; transcription factor activity; DNA binding response to amino acid; response to stress.
**MIR5770**	peroxiredoxin Q	response to oxidative stress.
	Calcium dependent protein kinase	Plant pathogen interaction protein kinase activity; hormone mediated signaling pathway; phosphate containing compound metabolic process.
**MIR6024**	abscisic acid 8'hydroxylase	oxidoreductase activity; response to fungus; response to light stimulus; sesquiterpenoid metabolic process; response to stress.
**MIR6164**	auxin regulated protein	Membrane.
	NAC domain protein	DNA binding.
	BI1 protein	fatty acid metabolic process; apoptotic process; response to stress; ER nucleus signaling pathway.
**MIR6485**	ethylene responsive transcription factor ERF054	Plant hormone signal transduction; DNA binding.
**MIR7539**	MYB transcription factor MYB54	nucleic acid binding; response to UV; response to acid; regulation of cellular metabolic process.
	Abscisic acid receptor PYL1	Plant hormone signal transduction; protein dimerization activity; isoprenoid binding regulation of phosphor protein phosphatase activity.
	Salt tolerance protein	transition metal ion binding; response to abiotic stimulus; response to organonitrogen compound; response to hormone.
	auxin induced protein X15	Plant hormone signal transduction; radial pattern formation; hormone transport; growth; gravitropism.
	abscisic acid receptor PYL9 isoform X1	Plant hormone signal transduction.
	auxin responsive protein	hormone mediated signaling pathway; postembryonic development; cell fate specification.
	Cell division cycle and apoptosis regulator protein	metal ion binding; programmed cell death.
	ethylene response sensor 1 isoform X1	Plant hormone signal transduction; protein histidine kinase activity; protein phosphorylation; signal transduction.
	myb transcription factor 1	nucleic acid binding response to UV; response to hormone; response to osmotic stress.
	ethylene responsive transcription factor SHINE 2	intracellular membrane bounded organelle; nucleic acid binding; transcription factor activity; DNA binding.
	ethylene responsive transcription factor RAP210	transcription factor activity; cell death; response to bacterium; response to organonitrogen compound; response to stress; nucleic acid binding.
**MIR9493**	zinc finger CCCH domain containing protein 44	intracellular membrane bounded; organelle transition; metal ion binding; nucleic acid binding.
**novel_mir_43**	zinc finger CCCH domain containing protein 38	cation binding.
**novel_mir_49**	WRKY transcription factor 22	Plant pathogen interaction.
	auxin response factor	Plant hormone signal transduction.
	auxin induced protein X15	Plant hormone signal transduction; radial pattern formation; hormone transport; growth; gravitropism.
	abscisic acid receptor PYL9 isoform X1	Plant hormone signal transduction.
	Cell division cycle and apoptosis regulator protein	metal ion binding; programmed cell death.
	ethylene response sensor 1 isoform X1	Plant hormone signal transduction; protein histidine kinase activity; protein phosphorylation; signal transduction.
	myb transcription factor	nucleic acid binding; response to UV; response to hormone; response to osmotic stress.
	ethylene responsive transcription factor SHINE 2	intracellular membrane bounded organelle; nucleic acid binding; transcription factor activity; DNA binding.
	Ethylene receptor	Plant hormone signal transduction; signal transducer activity.
	MYB transcription factor MYB127	nucleic acid binding; transition metal ion binding.
	WRKY transcription factor 40 isoform 1	response to bacterium; response to organonitrogen compound; response to acid; regulation of defense response to virus.
**novel_mir_4**	WRKY transcription factor 22	Plant pathogen interaction.
	Salt tolerance protein	transition metal ion binding; response to abiotic stimulus; response to organonitrogen compound; response to hormone.
	auxin induced protein X15	Plant hormone signal transduction; radial pattern formation; hormone transport; growth; gravitropism.
	abscisic acid receptor PYL9 isoform X1	Plant hormone signal transduction.
	auxin responsive protein	intracellular membrane bounded organelle; protein binding; hormone mediated signaling pathway.
	ethylene response sensor 1 isoform X1	Plant hormone signal transduction; protein histidine kinase activity; protein phosphorylation; signal transduction.
	myb transcription factor 1	nucleic acid binding response to UV; response to hormone; response to osmotic stress.
	NAC domain containing protein 8 isoform X1	DNA binding.
	auxin response factor 4	protein binding; hormone mediated signaling pathway; postembryonic development; cell fate specification.

### Key miRNAs in the *C*. *korshinskii* water stress response

**Fig 5:**
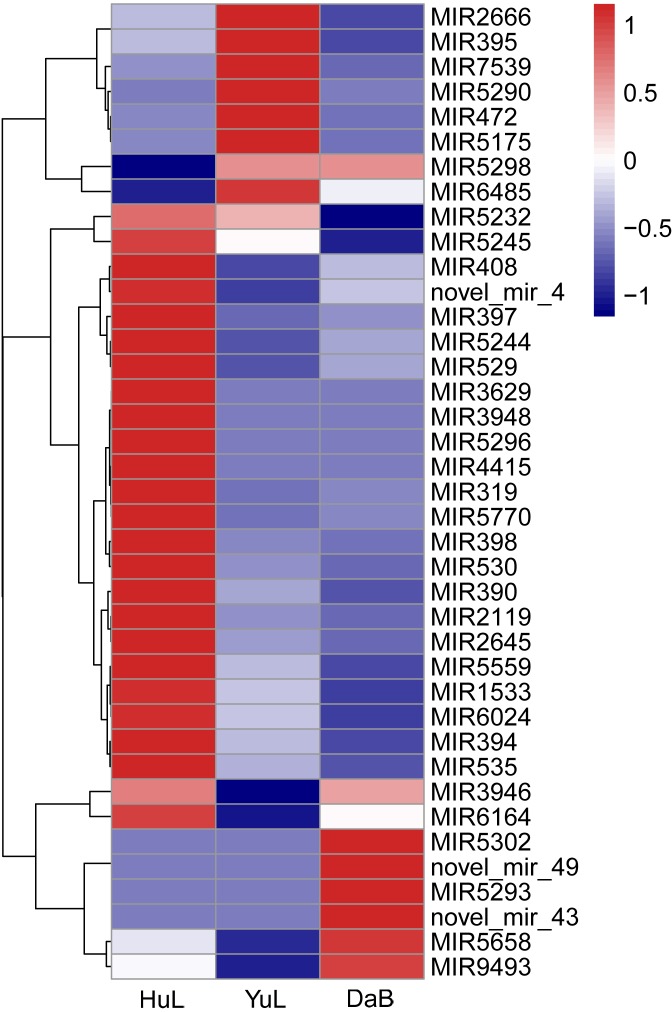
Differential expression profiles of drought-responsive miRNAs of *Caragana korshinskii* in Huangling (HuL), Yulin (YuL), and Dalad Banner (DaB).

**Fig 6:**
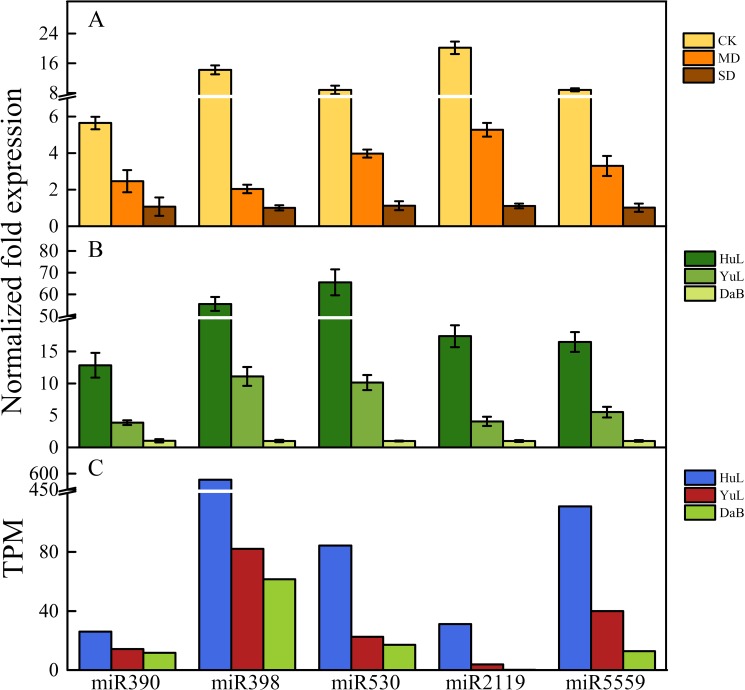
Expression levels of five miRNAs (miR390, miR398, miR530, miR2119, and miR5559) of *Caragana korshinskii* under laboratory and natural conditions and their sequencing data. (A) Relative expression levels of miR390, miR398, miR530, miR2119, and miR5559 in the control (CK), mild drought (MD), and severe drought (SD) samples. Relative expression levels are shown as fold changes, with the standard errors (SE) of three biological replicates. (B) Relative expression levels of miR390, miR398, miR530, miR2119, and miR5559 in the Huangling (HuL), Yulin (YuL), and Dalad Banner (DaB) samples. Relative expression levels are shown as fold changes, with the standard errors (SE) of three biological replicates. (C) Expression levels of sequencing data of miR390, miR398, miR530, miR2119, and miR5559 in the HuL, YuL, and DaB samples.

Regarding the gradient of moisture stress among *C*. *korshinskii* habitats, we compared the results from the laboratory water stress experiment and natural habitat. Combining the GO and KEGG annotation results (Table 2), we further carried out a trend analysis on the 39 miRNAs (Fig 5), based on a decrease in precipitation from HuL to DaB. Five putative drought-related miRNAs (miR390, miR398, miR530, miR2119, and miR5559) were selected for the stem-loop qRT-PCR analysis because their target genes were involved in abiotic stress-related processes, such as oxidative stress, auxin-response, cell death, and senescence. Using qRT-PCR, we measured the expression of these miRNAs in the laboratory- and field-grown samples. We found that the genes targeted by the 5 selected putative drought-related miRNAs (miR390, miR398, miR530, miR2119, and miR5559) exhibited a strong response to changes in soil moisture, and the expression of the five conserved miRNAs was downregulated by water stress under both field and laboratory conditions, which well matched the transcripts per million mapped reads results from the sequencing analysis (Fig 6). Although additional studies are needed to elucidate the function of these miRNAs in the stress-resistance of *C*. *korshinskii*, the consistency of our observations in both field-collected and laboratory-grown samples supports the role of the five identified miRNAs in the moisture response of *C*. *korshinskii* along the precipitation gradient from HuL to DaB.

### Key target genes and miRNA of *C*. *korshinskii* collaborate in responding to water stress

**Fig 7:**
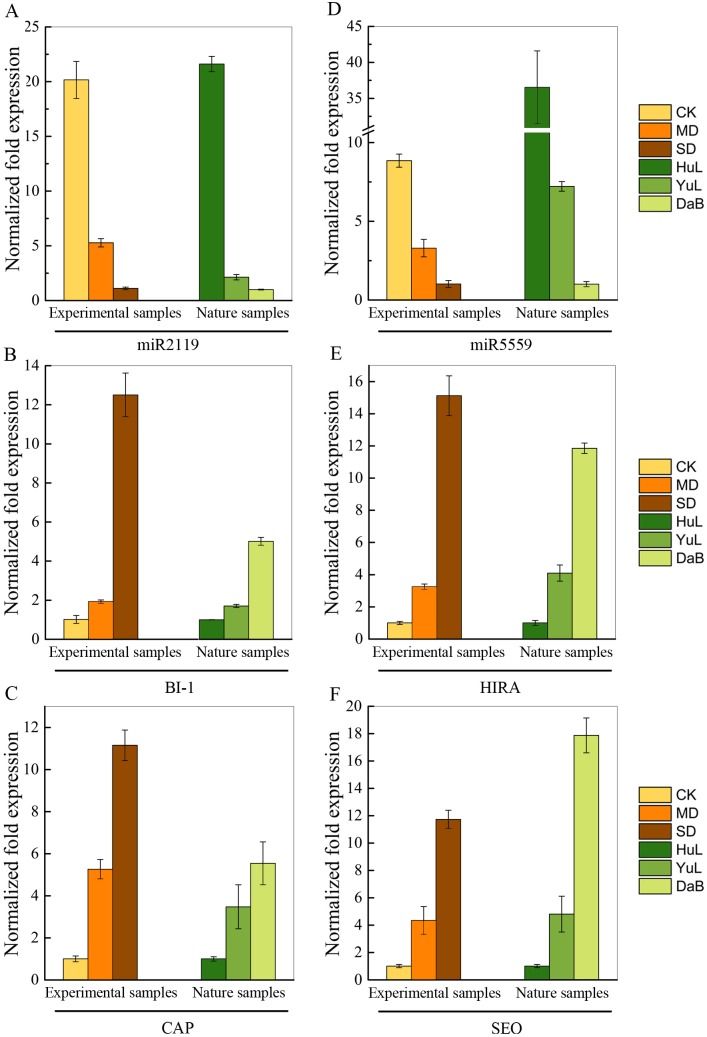
Expression analysis (qRT-PCR) of miRNA target genes of *Caragana korshinskii* under wild and laboratory conditions. (A, D) miR2119 and miR5559 expression under wild and laboratorial conditions. (B, C) qRT-PCR result of predicted target genes of miR2119 under wild and laboratory conditions. (E, F) qRT-PCR result of predicted target genes of miR5559 under wild and indoor conditions. Relative expression levels are shown as fold changes, with the standard errors (SE) of three biological replicates.

To further validate the mechanism of the miRNA response to drought, we selected two unauthenticated miRNAs (miR2119 and miR5559) to investigate their target genes (S4 Table). We found that the Bax inhibitor-1 (*BI-1*) and Clathrin assembly protein (*CAP*) genes were potential targets of miR2119, whereas the histone regulation (*hira*) and sieve element occlusion (*SEO*) genes were potential targets of miR5559. Using qPCR, we found that the expression of the 4 genes in both the field-collected and laboratory-grown samples was significantly upregulated as a result of a water deficit effect, which suggested that there was a negative correlation between miR2119 and miR5559 and their specific target genes (Fig 7). Therefore, the 4 genes (*BI-1* and *CAP*; *HIRA* and *SEO*), collaborating with their respective miRNAs (miR2119; miR5559), were involved in the stress response in *C*. *korshinskii*, where further study would bring more information on the functions of the 2 miRNAs.

### Validation of *C*. *korshinskii* miRNAs by qRT-PCR

**Fig 8:**
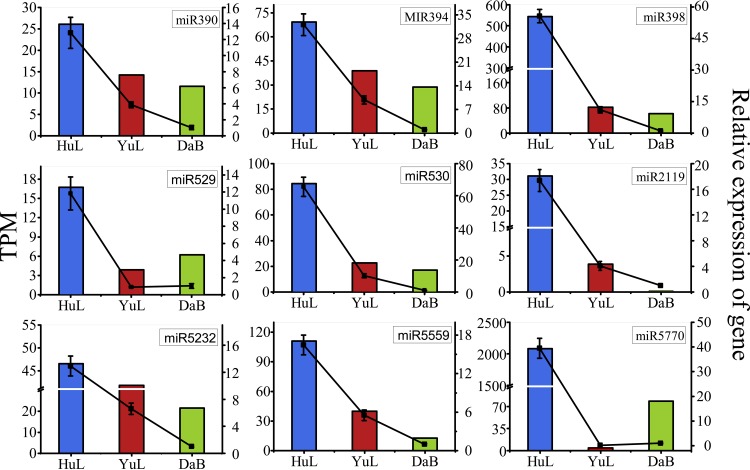
Expression analyses of selected miRNAs of *Caragana korshinskii* as evaluated by qRT-PCR. The relative expression levels are shown as fold changes with the standard errors (SE) of three biological replicates.

Stem-loop qRT-PCR confirmed the sequencing results and indicated that the 9 representative known miRNAs (miR390, miR394, miR398, miR529, miR530, miR2119, miR5323, miR5559, and miR5770) were differentially expressed among the 3 samples (HuL, YuL, and DaB; Fig 8).

## Discussion

Drought stress is one of the major natural constraints on plant growth and development. As an important species for preventing desertification in the Loess Plateau, *C*. *korshinskii* is well adapted to water shortage, and even modifies its gene expression and morphology under drought conditions. For instance, our previous work revealed that the size and density of *C*. *korshinskii* trichomes are correlated with the precipitation gradient that occurs across the Loess Plateau, China [22]. However, the underlying regulatory mechanism of this species’ adaptation to natural drought conditions remains poorly understood. In the present study, we generated a large amount of miRNA expression data from *C*. *korshinskii* using the high-throughput Solexa sequencing technology. By deep sequencing *C*. *korshinskii* small RNAs and aligning them to known plant miRNAs, we identified 490 known miRNAs, as well as 96 novel miRNAs. MiRNAs have been implicated in the regulation of plant responses to several types of biotic and abiotic stress. Some of the known miRNAs have been detected in other plant groups, whereas others are unique to legumes [35]. Thus, our study provides a foundation for further research into the miRNA-mediated gene regulation of stress responses in *C*. *korshinskii*.

The main aim of this study was to investigate the miRNAs of *C*. *korshinskii*, particularly those related to drought tolerance. By analyzing the functions of putative target genes using the GO analysis, we selected and screened 39 miRNAs with biological functions that were possibly related to drought tolerance (Table 2). The targets of these miRNAs included genes involved in fatty acid metabolism, apoptotic process, programmed cell death, plant hormone signal transduction, and responses to osmotic and oxidative stress. The variance in miRNA expression among the sampling sites may contribute to the differential regulation of these target genes.

Many miRNAs were down- or upregulated in response to the precipitation gradient (Fig 5), and some of them have been shown to respond to biotic or abiotic stress by previous studies. For example, miR5244 and miR5232 have been reported to accumulate in response to arbuscular mycorrhizal (AM) symbiosis in *M*. *truncatula* roots [36]. In addition, in *Arabidopsis*, miR390, TAS3 tasiRNAs, and their auxin response factor targets constitute an autoregulatory network that quantitatively regulates lateral root growth; lower levels of the miR390 transcript facilitate the expression of auxin response factor genes, thus promoting the development of strong root systems and drought tolerance. In addition, miR530 is highly upregulated in response to fungal infection in chickpea [35], and its target genes vary under different conditions and in different plant species and tissues. One of the target genes of miR530 is a tandem zinc knuckle/PLU3 domain encoding gene (At5g43630; *TZP*), which is involved in the regulation of morning-specific growth in *Arabidopsis* [37]. miR398 targets two Cu/Zn superoxide dismutases [Cu/Zn Superoxide dismutase 1 (CSD1), and Cu/Zn Superoxide dismutase 2 (CSD2)] in *Arabidopsis thaliana* [38] and is downregulated in response to both biotic (*Pseudomonas syringae*) and abiotic stress (ozone and salinity) [39]. Experimental results indicated that CSD1 mRNA levels were found to be negatively correlated with miR398 levels during exposure to ozone, salinity, and biotic stress, which suggests that a link exists between oxidative stress and miR398 [39]. The evidence gathered by another study indicated that miR398 regulates the responses of rice to a wide range of environmental stress and to ethylene, and that miR398 functions by mediating the expression of CSDs (Cu/Zn superoxide dismutases) and cellular levels of reactive oxygen species [40]. MiR319 has been demonstrated to target *TCP* [for TEOSINTE BRANCHED/CYCLOIDEA/PROLIFERATING CELL FACTORS (PCF)] genes, which are involved in cell differentiation, leaf development, senescence, and the biosynthesis of jasmonic acid [32,41,42]. The miR319-mediated downregulation of target genes (*TCPs*) in overexpressing omiR319 transgenic plants causes changes in various biological processes, including those associated with water retention capacity, leaf wax synthesis, and salt uptake [32]. The prediction of potential target genes for the conserved and novel miRNAs was based on sequence homology [35], since many miRNAs respond similarly under both biotic and abiotic stress. The existence of crosstalk between the pathways that are involved in regulating stress indicates that miRNAs that respond to the precipitation gradient may be responsible for the adaptive mechanisms of wild *C*. *korshinskii* on the Loess Plateau [35].

Moreover, our finding that some key conserved miRNAs respond to water stress in *C*. *korshinskii* agrees with previous studies. During drought stress, the levels of miR408 transcripts decrease significantly in rice, whereas they remain elevated in tolerant rice [43]. By predicting the target genes of osa-miR408 in rice, it was determined that osa-miR408 regulates members of the plastocyanin-like protein family. Phytocyanins (PCs) are ancient blue copper proteins that can bind to a single copper atom, function as electron transporters, and are involved in responses to drought stress [43]. MiR408 was also downregulated by drought stress in *Populus* [44]. MiR394 was identified among the stress-responsive microRNAs of *Populus* [44,45] and was downregulated under drought conditions [44]. The predicted targets of miR394 are annotated as dehydration-responsive and F-box proteins, which were recently reported to be differentially regulated under stressful conditions and to play significant roles in the abiotic stress response pathway.

The identification of miRNA target genes is critical for understanding their functional implications. Of the miRNAs that were down- or upregulated by the precipitation gradient, miR2119 and miR5559 have only been reported in legumes, and their function is still unclear. By computational analysis of the transcriptome data, *BI-1* and *CAP* were predicted as the targets of the miR2119. *BI-1*, a widely conserved cytoprotective protein that is localized in the endoplasmic reticulum membrane, is an attenuator of both biotic and abiotic types of cell death [46,47]. Moreover, CAP is a key protein in the formation of clathrin-coated pits, which are an intermediate in the process of clathrin vesicle formation [48]. *HIRA* and *SEO* are the predicted target genes of miR5559. *HIRA* is involved in cell senescence via a pathway that appears to depend on the flux of heterochromatic proteins through promyelocytic leukemia bodies [49], while *SEO* genes, which encode forisome subunits (structural phloem proteins unique to the sieve elements of legumes), have been identified in *M*. *truncatula* and other legumes [48].

To support our analysis of wild *C*. *korshinskii*, we designed an indoor experiment to detect the expression of the 5 miRNAs (miR390, miR398, miR530, miR2119, and miR5559) in response to reduced soil moisture. Under water stress, the expression of the 5 miRNAs was consistent with both the sequencing and qPCR analyses of the wild plant samples. Furthermore, the expression of *BI-1* and *CAP*, as well as *HIRA* and *SEO*, the predicted target genes of miR2119 and miR5559, respectively, were measured for both the wild and indoor-grown plants. Although we found that the expression levels of the miRNAs and their target genes were negatively correlated, indicating that key miRNAs could be involved in water stress responses, further experiments are needed to elucidate interactions of miRNAs with their predicted targets and their regulatory mechanisms in physiological processes.

The predicted targets for the novel miRNAs were involved in plant growth, development, stress response, hormone signal transduction, and transcription. With a major concern on responding to the precipitation gradient on the Loess Plateau, novel_mir_43 was predicted to target the WRKY transcription factor, which is essential in the pathogen and salicylic acid responses of higher plants, as well as in a variety of other plant-specific reactions [50,51]. One study found that the WRKY transcription factor was differentially regulated in plants that were exposed to abiotic stress factors such as NaCl, polyethylene glycol, cold, and heat stress [52]; another reported that the transcription factor enhanced drought tolerance by retarding leaf wilting and increasing the survival rate of green plant parts [53]. Putative targets were also predicted for novel_mir_49, including the WRKY transcription factor, Na+/H+ antiporter, zinc finger CCCH domain-containing protein, and a series of molecules involved in plant hormone signal transduction, such as the auxin response factor, abscisic acid receptor, and ethylene responsive transcription factor. Because they clearly responded to the precipitation gradient on the Loess Plateau, the validity of these novel miRNAs deserves further investigation, in order to confirm their differential expression, which will provide clues about their biological functions.

## Conclusions

Using deep-sequencing technology, we identified 490 known miRNAs and 96 novel miRNAs in *C*. *korshinskii* sampled from the Loess Plateau, including 39 miRNAs that may be associated with drought response. After analyzing the response of the miRNAs to a precipitation gradient, we investigated the expression of 5 key miRNAs under both natural and experimental water stress conditions. Our results provide a better understanding of the functional relationships and specific miRNAs involved in the stress-related gene regulatory pathways. Additional work is needed to further elucidate this relationship, utilizing the available unique datasets in *C*. *korshinskii*. Further characterization of the targets of drought-associated miRNAs will also help understand the drought response and the tolerance of *C*. *korshinskii*, which serves as an important model for stress resistance in plants facing desertification.

## Supporting information

S1 Table List of nine miRNA primers used for qPCR analysis. (DOCX)

S2 Table List of identified known miRNAs in the three libraries. (XLSX)

S3 Table List of identified novel miRNAs in the three libraries. (XLSX)

S4 Table List of results of annotation for known and novel miRNAs. (XLSX)
