# Supplementary material for: Unraveling the microRNA of Caragana korshinskii along a precipitation gradient on the Loess Plateau, China, using high-throughput sequencing
Source: PLoS One. 2017 Feb 16;12(2):e0172017. doi: 10.1371/journal.pone.0172017 (PMC5313209; doi:10.1371/journal.pone.0172017)
Supplement: S1 Table — (DOCX) [file pone.0172017.s001.docx]

**Table**

|  | **Supplemental Table 1** list of nine miRNA primers used for qPCR analysis. | | | |
| --- | --- | --- | --- | --- |
|  | | | | |
|  | **miRNA** | **miRNA sequence (5'-3')** | **Primer** | **Primer Sequence (5'-3')** |
|  | MIR390 | CGCTATCCATCCTGAGTTTCA | RT primer | GTCGTATCCAGTGCAGGGTCCGAGGTATTCGCACTGGATACGACTGAAAC |
|  |  |  | Forward primer | GCGGCGGCGCTATCCATCCTGAG |
|  | MIR394 | TTGGCATTCTGTCCACCTCC | RT primer | GTCGTATCCAGTGCAGGGTCCGAGGTATTCGCACTGGATACGACGGAGGT |
|  |  |  | Forward primer | GCGGCGGTTGGCATTCTGTCCAC |
|  | MIR398 | TGTGTTCTCAGGTCGCCCCTG | RT primer | GTCGTATCCAGTGCAGGGTCCGAGGTATTCGCACTGGATACGACCAGGGG |
|  |  |  | Forward primer | GCGGCGGTGTGTTCTCAGGTCG |
|  | MIR529 | TTGACAGAAGAGAGAGAGCAC | RT primer | GTCGTATCCAGTGCAGGGTCCGAGGTATTCGCACTGGATACGACGTGCTC |
|  |  |  | Forward primer | GCGGCGGTTGACAGAAGAGAGAG |
|  | MIR530 | TGCATTTGCACCTGCACTTTA | RT primer | GTCGTATCCAGTGCAGGGTCCGAGGTATTCGCACTGGATACGACTAAAGT |
|  |  |  | Forward primer | GCGGCGGTGCATTTGCACCTGC |
|  | MIR2119 | TCAAAGGGAGGTGTGGAGTAG | RT primer | GTCGTATCCAGTGCAGGGTCCGAGGTATTCGCACTGGATACGACCTACTC |
|  |  |  | Forward primer | GCGGCGGTCAAAGGGAGGTGTG |
|  | MIR5232 | CGTGTCGCTCTCACCTGGAGC | RT primer | GTCGTATCCAGTGCAGGGTCCGAGGTATTCGCACTGGATACGACGCTCCA |
|  |  |  | Forward primer | GCGGCGGCGTGTCGCTCTCACC |
|  | MIR5559 | TACTTAGTGAATTGTTGGATC | RT primer | GTCGTATCCAGTGCAGGGTCCGAGGTATTCGCACTGGATACGACGATCCA |
|  |  |  | Forward primer | GCGGCGGTACTTAGTGAATTGTTG |
|  | MIR5770 | TAGGACTATGGTTTGGACGAT | RT primer | GTCGTATCCAGTGCAGGGTCCGAGGTATTCGCACTGGATACGACATCGTC |
|  |  |  | Forward primer | GCGGCGGTAGGACTATGGTTTG |
|  | Reverse primer: ATCCAGTGCAGGGTCCGAGG | | | |
